# Transforming a Young Life: Grade 5 Schwab Osteotomy for a Child With Congenital Kyphoscoliosis With Rare T12 Hemivertebra

**DOI:** 10.7759/cureus.74146

**Published:** 2024-11-21

**Authors:** Imran F Gul, Fadzrul Abbas Mohamed Ramlee, Mohd Hezery Harun, Teck Siang Lim

**Affiliations:** 1 Orthopedic Surgery, Universiti Putra Malaysia, Serdang, MYS

**Keywords:** congenital kyphoscoliosis, hemivertebrae, schwab osteotomy, spinal deformities, turner’s syndrome

## Abstract

Kyphoscoliosis is a spinal disorder where the spine’s natural curvature is abnormally altered in multiple planes. It may be associated with the presence of a hemivertebrae, as discussed in this case. Negligence of this deformity may cause symptoms of back pain, worsening spinal deformity leading to reduced lung expansion, or neurological deficit impacting the quality of life for the worse. The aim of surgery is to correct the spinal deformity. In this case, a Schwab Grade 5 osteotomy was performed along with instrumentation and fusion of the adjacent vertebrae. It is important that thorough preoperative planning is performed before the surgery, as complications may arise from the Schwab Grade 5 osteotomy procedure resulting in lifelong disability. Refined osteotomy techniques could be explored to reduce the risk of surgical complications in the near future.

## Introduction

Kyphoscoliosis is a spinal condition characterized by an abnormal curvature of the spine in both the coronal and sagittal planes. It is a combination of kyphosis and scoliosis, leading to the spine curving abnormally on the sagittal plane, causing a rounded or hunched appearance, and on the coronal plane, resulting in a sideways twist [1]. This condition can occur at any age and is often idiopathic with symptoms ranging from a hunched back to more severe effects on the lungs and heart. Hemivertebra is a common cause of congenital scoliosis [1]. When the hemivertebra is fully or semi-segmented, progression of the curve is usually unavoidable. This means that individuals with congenital scoliosis caused by hemivertebra are more likely to experience a worsening of their spinal curvature over time. The severity of the deformity and the number of hemivertebrae present can impact the rate of deterioration. In the treatment of congenital kyphoscoliosis, various surgical procedures are used, such as combined anterior and posterior convex hemiepiphysiodesis and excision of the hemivertebra via posterior or anterior-posterior fusion techniques [2]. However, treating severe spinal deformities in children is highly challenging. Procedures such as in-situ fusion or hemiepiphysiodesis at a young age can slow down or stop growth, achieving limited correction rates [2,3]. The definitive treatment for congenital kyphoscoliosis caused by hemivertebra should involve the removal of the hemivertebra. The Schwab Grade 5 osteotomy is a surgical technique employed for the correction of significant spinal deformities. The osteotomy involves cutting and repositioning the entire vertebrae and adjacent discs to improve the alignment of the spine. This type of surgery aims to correct the deformity, reduce pain, and improve the overall function of the spine [2].

## Case presentation

**Figure 1:**
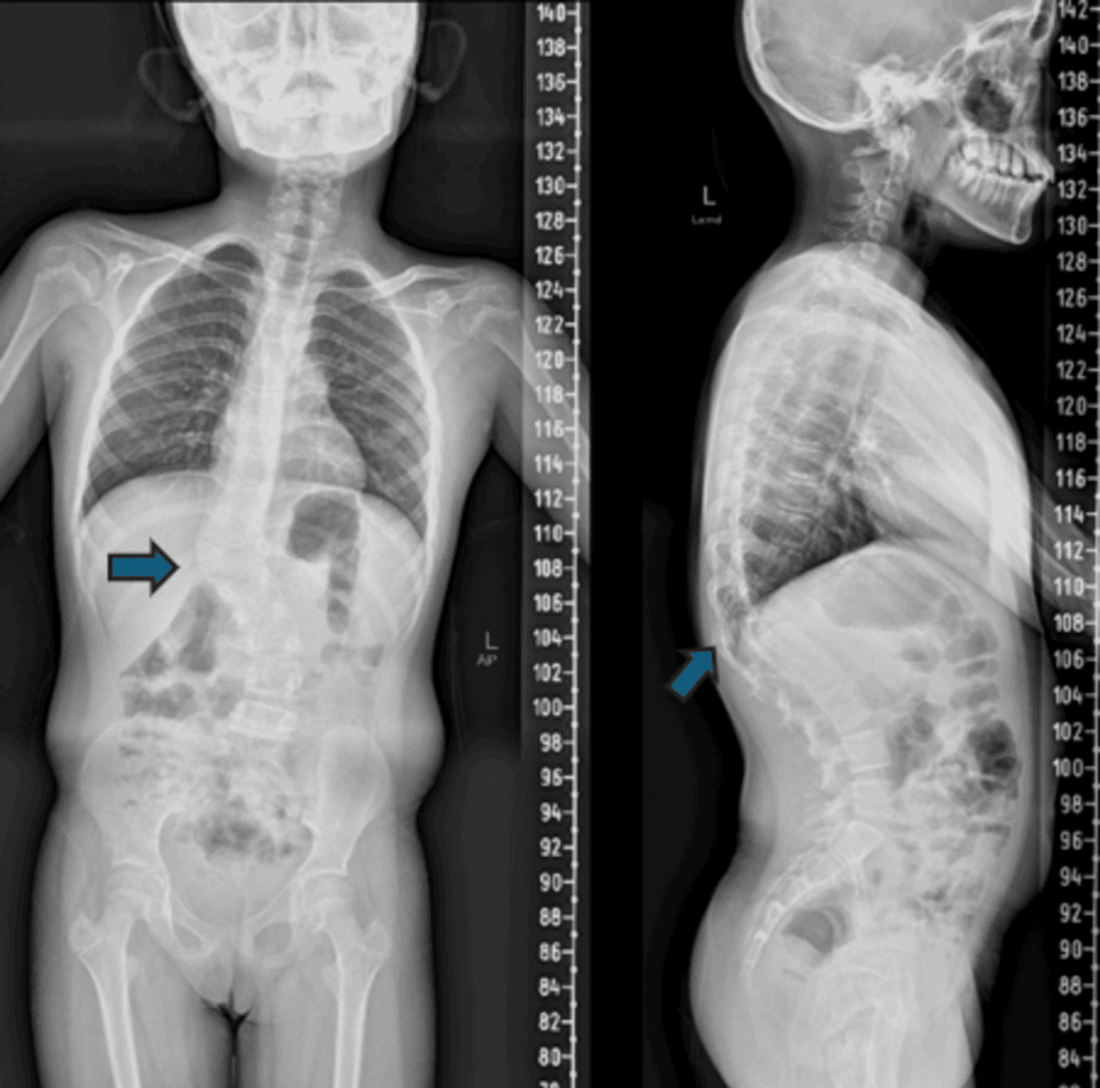
Anteroposterior and lateral views of the whole spine. X-ray shows thoracolumbar scoliosis with kyphotic deformity primarily at the T12 vertebra. Cobb’s angle of 43 degrees from T10 to T12 and Cobb’s angle of 41 degrees from L1 to L4 can be seen. The kyphosis angle of 52.5 degrees can be noted from T11 to L1.

**Figure 2:**
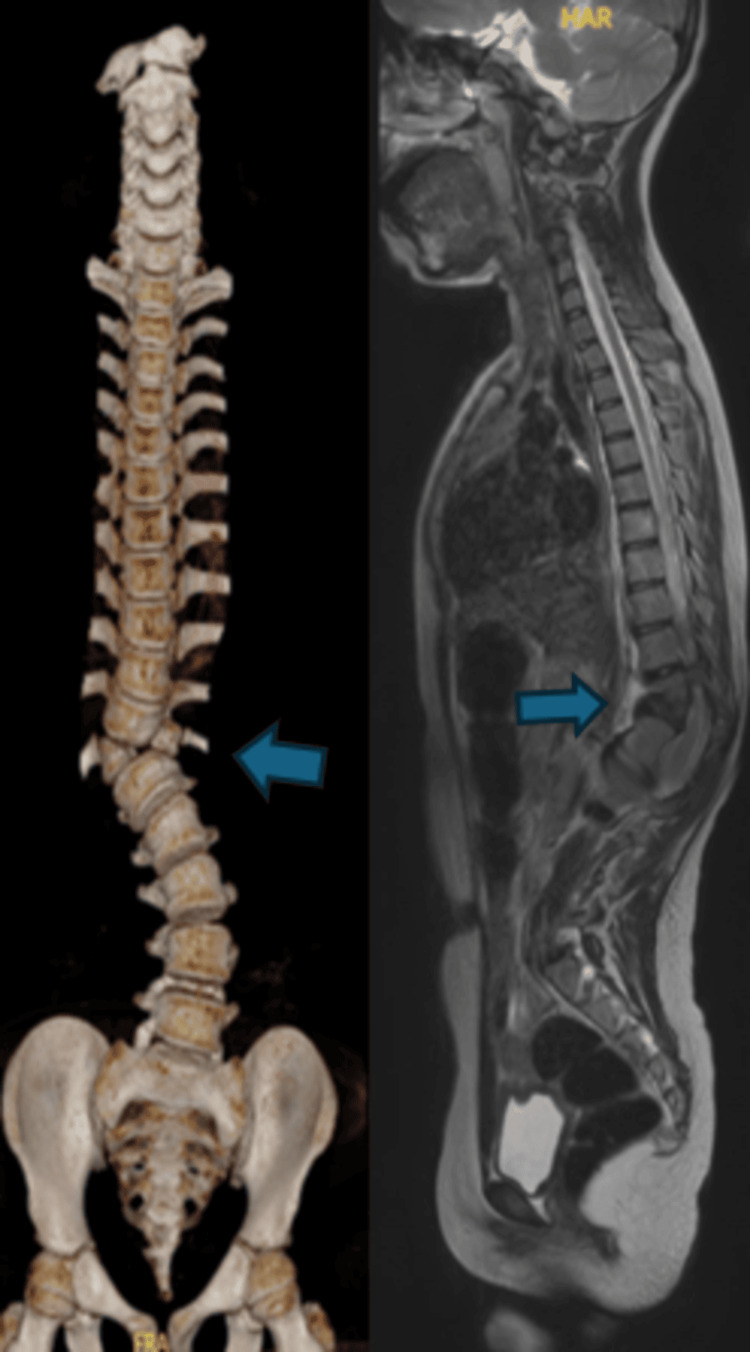
Three-dimensional computed tomography image (left) of the whole spine in coronal view showing lumbar levoscoliosis with T12 hemivertebra. Magnetic resonance imaging (right) of the whole spine in sagittal view showing thoracolumbar dextroscoliosis with butterfly vertebrae of T12.

An 11-year-old girl, diagnosed with Turner syndrome presented with spine deformity. It was first realized when she was five years old when her mother noticed her daughter’s shoulders were uneven. As the patient grew older, her spine deformity became more noticeable. The patient, however, did not complain of any back pain, muscular weakness, breathlessness, or numbness. On examination, upon inspection from the back, there was a right shoulder tilt, the right scapula was more noticeable, and a slight right lateral pelvic tilt was present. Lateral inspection showed hyperkyphosis of the lower thoracic region. Upon forward bending, there was an obvious right thoracic hump. No neurocutaneous signs were seen. Motor and sensory functions were otherwise intact. On a whole spine X-ray, there was obvious thoracolumbar scoliosis seen with Cobb’s angle of 43 degrees from T10 to T12 and Cobb’s angle of 41 degrees from L1 to L4. The kyphosis angle was 52.5 degrees from T11 to L1 (Figure 1). A computed tomography scan of the spine showed lumbar levoscoliosis with T12 hemivertebrae (Figure 2). Magnetic resonance imaging of the whole spine showed thoracolumbar dextroscoliosis with butterfly vertebrae of T12 (Figure 2). Corrective deformity surgery along with Schwab Grade 5 osteotomy of T12 was planned for the patient.

**Figure 3:**
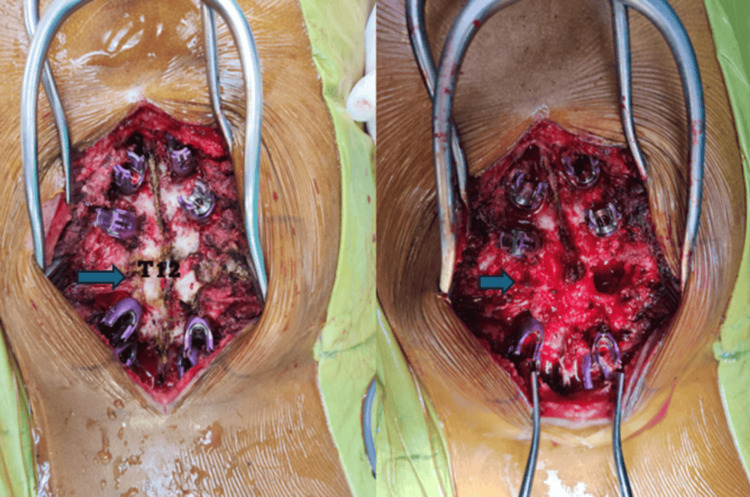
Intraoperative image on the left shows pedicle screws placed with T12 still intact. The image on the right shows post-T12 vertebra resection.

**Figure 4:**
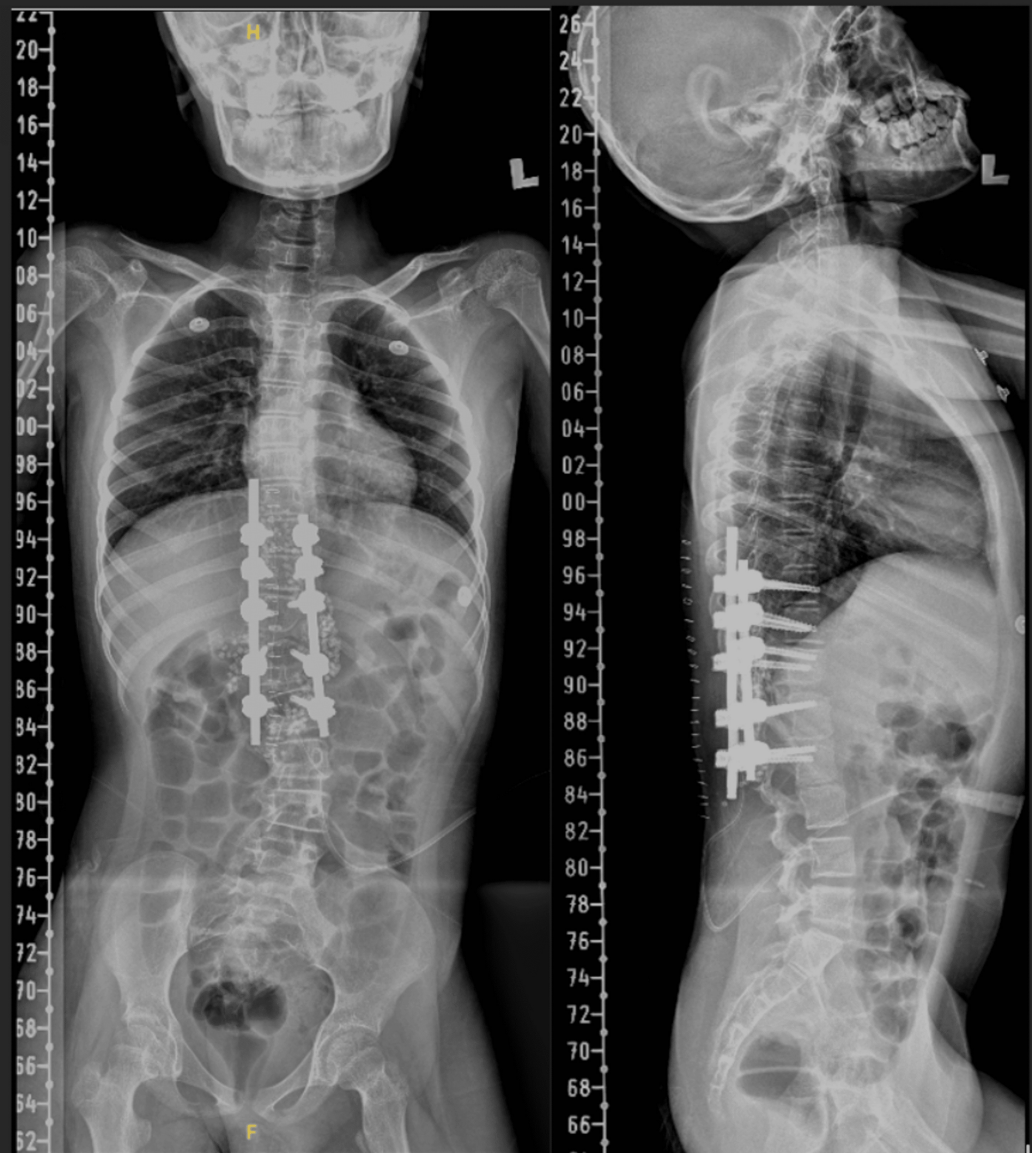
Postoperative X-rays of the whole spine depicting that the spinal deformity has been well corrected with the absence of the T12 vertebra.

Before starting the surgery, the T12 was marked. Pedicle screws were inserted from T9 to L2 except for T12 vertebra. Intraoperatively, the spinal cord, traversing nerve root of L1, and exiting nerve root of T12 were protected. The T12 vertebral body was visualized, and seen as hemivertebrae (Figure 3). A Grade 5 osteotomy was then performed including resection of the rib cage articulating to the T12 transverse process (Figure 3). The short segmental rod on the concave side was kept aside to preserve the correction at the apical vertebrae. A lengthy rod was inserted on the convex side spanning from the upper instrumented vertebra to the lower instrumented vertebra. The rod cantilever technique was then applied to enhance the correction of both scoliosis and kyphosis, resulting in the achievement of balance in both the coronal and sagittal planes (Figure 4). Throughout the surgery, there was no deterioration or loss in signal of the neuromonitor. Postoperatively, the patient was monitored in the intensive care unit for a day. X-rays post-surgery showed that the deformity had been corrected. There were no neurological deficits upon post-surgical neurological assessment. The patient was subsequently discharged home. There were no complications identified during clinic follow-ups.

## Discussion

The natural progression of congenital spinal deformity is known to be continuously worsening, leading to serious consequences. Quick surgical interventions seem to be the most effective way to manage kyphoscoliosis deformity, and it is essential to precisely perform osteotomy on each patient’s spinal deformity to ensure a consistently stable spinal position in the long run. There is a general consensus that efforts should be made to minimize the number of vertebrae resected, as a higher level of resection is linked to an increased risk of permanent neurological complications [2,4]. Correcting severe and rigid kyphoscoliosis through surgery is challenging with high risks of neurological injury, permanent paralysis, and even death [5]. Various factors, such as age, cause of the condition, the extent of spinal deformity, spinal cord function, the specific surgical techniques used, and the rate at which the deformity is corrected, are known to contribute to the likelihood of experiencing neurological complications. In this case, a singular complete vertebral and disc resection (Schwab Grade 5 osteotomy) was the preferred choice as a singular vertebra, i.e., the T12 vertebra, was the apex of the kyphoscoliosis deformity. By addressing the T12 vertebra, it was possible to achieve optimum correction of the deformity and sagittal balance. The patient will need postoperative rehabilitation in the form of physiotherapy for back muscle strengthening and posture training. A common complication that may arise from corrective deformity surgery in a pediatric patient is the crankshaft phenomenon where there is gradual development of rotational and angular spinal deformity that can occur after undergoing surgery on the back of the spine [6]. In skeletally immature patients, the crankshaft phenomenon is thought to occur because the front parts of the spine continue to grow even after a solid fusion has been achieved in the back during surgery [6]. During the patient’s follow-up, we need to be vigilant so as to not miss the aforementioned complication.

## Conclusions

The Schwab Grade 5 osteotomy is a specific kind of spinal surgery used to correct severe and rigid kyphoscoliosis, which is a condition involving both forward and sideways curvature of the spine. This procedure is part of a step-by-step correction plan. While it is a more aggressive approach with a higher risk of complications compared to other types of osteotomies, it can be particularly effective in dealing with the challenges of severe and rigid spinal deformities. The decision to opt for this procedure involves careful consideration of the patient’s condition and the potential benefits weighed against the associated risks.
